# Monoaminylation in Human Health and Disease: State of the Field, Challenges, and Emerging Directions

**DOI:** 10.1002/advs.202520653

**Published:** 2026-02-09

**Authors:** Yiqi Zhao, Hongli Zhang, Yating Yang, Wei‐Dong Chen, Yan‐Dong Wang

**Affiliations:** ^1^ State Key Laboratory of Chemical Resource Engineering College of Life Science and Technology Beijing University of Chemical Technology Beijing P. R. China; ^2^ Key Laboratory of Receptors‐Mediated Gene Regulation and Drug Discovery School of Basic Medical Science Inner Mongolia Medical University Hohhot Inner Mongolia P. R. China

**Keywords:** diseases, histone, monoaminylation, post‐translational modification

## Abstract

Parallel to their roles in regulating receptor activation, mounting evidence suggests that monoamines are involved in protein post‐translational modification, which regulates the physiological function of the nervous system and various pathophysiologically relevant conditions. These modifications, primarily including serotonylation, dopaminylation, and histaminylation, occur on both histone and non‐histone proteins, thereby modulating gene transcription and protein function. This review comprehensively summarizes the types of monoaminylation, the catalytic enzymes and molecular mechanisms, and the corresponding monoamine transporters. Furthermore, we categorize and discuss the roles of monoaminylation across the human body in health and disease, highlighting their functional implications and therapeutic potential. We emphasize the need for precise methodologies to modulate monoamine levels in the context of pathological processes across various diseases. Finally, we outline persistent challenges in the field and suggest promising directions for future research, aiming to facilitate both mechanistic insight and translational applications.

## Graphical abstract

This review delineates monoaminylation—serotonylation, dopaminylation, and histaminylation—as key post‐translational modifications beyond receptor signaling. It details their catalytic mechanisms, roles in gene expression and protein function, and implications in health and disease, aiming to bridge mechanistic insights with therapeutic potential.

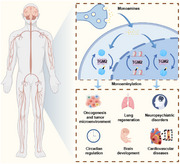

## 1 Introduction

Given that the nervous system is an essential regulator of both physiological functions and pathophysiological processes, it is therefore not surprising that it plays a pivotal role in modulating the human body in both health and disease [1]. Parallel to its roles in governing the growth, upkeep, and regeneration of diverse organs and tissues, the nervous system similarly co‐opts neural mechanisms within malignant cellular niches throughout the body, particularly in cancer [1, 2]. Recently, foundational discoveries have revealed that direct electrochemical communication occurs in several cancers, forming bona fide functional chemical synapses between cancer cells and neurons [3, 4, 5, 6, 7]. Additionally, another well‐known direct communication channel between neurons and non‐neuronal cells, via paracrine or endocrine mechanisms, is mediated by neurotrophins and neurotransmitters and occurs in nervous, developmental, and immune systems, as well as in cancers [8, 9, 10, 11, 12, 13, 14]. However, previous studies mainly focused on regulating neuronal signals at their corresponding receptors. Recently, a new class of works about monoamine modifications has extended the understanding of neurotransmitter function, indicating these neurotransmitters, including serotonin, dopamine and histamine, can directly entering the nucleus to covalently modify histones (termed as serotonylation, dopaminylation and histaminylation, respectively) via TGM2‐catalyzed isopeptide bonds, enabling a shift from “rapid” neural signaling to “long‐term” transcriptional regulation [15, 16, 17]. Moreover, parallel transamidation of non‐histone substrates establishes neuroproteomic regulation, suggesting that monoaminylation can be implicated in various physiological and pathological contexts [18]. Therefore, this neuro‐epigenetic and neuro‐proteomic coupling constitutes a distinctive neuronal regulatory circuit that is reshaping understanding of neural plasticity, tumor microenvironments, and systemic pathophysiology.

This review presents current knowledge of monoaminylation, providing a detailed overview of the types, functions, mechanisms, and catalytic enzymes involved, and summarizing monoamine transporters. Furthermore, we demonstrate recent empirical data showing the role of monoaminylation in health and disease. Finally, persistent technological barriers constrain the clarification of mechanisms, notably antibody cross‐reactivity and mass spectrometry sensitivity limits. We evaluate emerging detection strategies and map therapeutic opportunities arising from this neural‐chromatin‐proteome axis to expand the therapeutic potential of existing neuro‐modulatory drugs.

## 2 The Substrates and Enzymes Involved in Monoaminylation

As early as the last century, studies identified that monoamine neurotransmitters could be incorporated into protein substrates under the catalysis of calcium ion (Ca^2^^+^)‐dependent transglutaminases (TGMs) [19]. However, the concept of “monoaminylation” as a distinct post‐translational modification (PTM) was not formally proposed and defined until 2003 [20]. This crucial milestone arises from research on platelet activation and aggregation mechanisms, in which transglutaminase catalyzes the covalent binding of serotonin (5‐hydroxytryptamine) to small GTPases (such as RhoA and Rab4) via transamidation, thereby conferring constitutive activity upon these proteins [21]. This discovery reveals the role of serotonin as a modification substrate. It prompts subsequent studies confirming that other monoamine neurotransmitters (e.g., histamine, dopamine, and norepinephrine) can similarly modify protein substrates [22]. Notably, in 2019, researchers first reported H3Q5ser modification, revealing that serotonin exerts an epigenetic regulatory function in addition to its role as a neurotransmitter [15]. This foundational discovery establishes a direct molecular bridge between neural signaling and genomic regulation. In 2020, H3Q5dop was identified in the striatum, confirming its role in transcriptional programming linked to drug addiction [16]. Recently, a work from Zheng et al. (2025) reports the novel histone H3Q5his modification (H3Q5his) and systematically elucidates its core mechanism in regulating circadian rhythms in the brain [17]. The discovery of histone monoaminylation marks the entry of monoaminylation into the field of epigenetic regulation, uncovering novel regulatory mechanisms within the dynamic epigenetic landscape.

**FIGURE 1:**
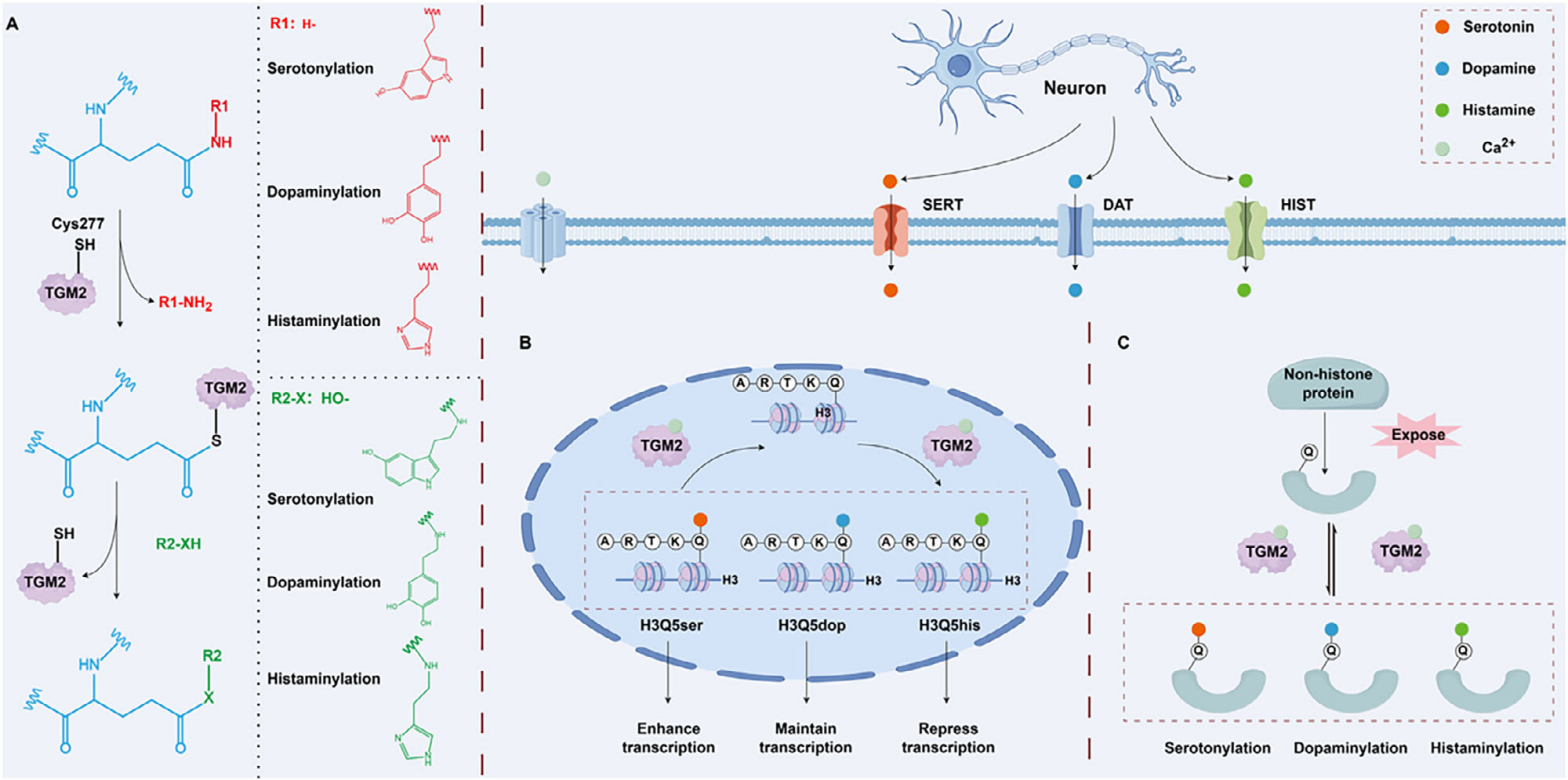
The mechanism of TGM2‐catalyzed monoaminylation. (A) The reversible process of monoaminylation is catalyzed by TGM2 using monoamine substrates, wherein the C277 residue of TGM2 acts as a nucleophile to attack glutamine residues on target proteins, resulting in the incorporation of monoamines via nucleophilic substitution. (B,C) Monoamine neurotransmitters secreted by neurons are taken up by cells via specific monoamine transporters. TGM2 utilizes these monoamines to catalyze monoaminylation on different protein substrates, including histone H3 and non‐histone proteins. TGM2: transglutaminase 2; SERT: serotonin transporter; DAT: dopamine transporter; HIS T: histamine transporter; H3Q5Ser: histone serotonylation; H3Q5Dop: histone dopaminylation; H3Q5His: histone histaminylation; Q: glutamine residues. We acknowledge the use of KingDraw software (KingDrawPc_V3.0.2.20) for drawing this figure.

Histone monoaminylation refers to the covalent attachment of monoamine neurotransmitters (e.g., 5‐hydroxytryptamine, dopamine, and histamine) to glutamine (Gln5) at position 5 of histone H3 via an isopeptide bond [23]. This results in the modifications of histone H3Q5ser (serotonylation), H3Q5dop (dopaminylation), and H3Q5his (histaminylation), all of which are specific manifestations of the transamidation reaction that covalently conjugates monoamines to glutamine residues across diverse protein substrates [24, 25, 26, 27] (Figure 1A). The diversity of monoamine modifications is reflected not only in the chemical properties of the modifying groups but also in their dynamic changes and functional differences: (1) H3Q5ser enhances transcriptional activation [28]; (2) H3Q5dop maintains persistent transcriptional programming [16]; (3) H3Q5his represses H3K4 methylation by antagonizing WD Repeat Domain 5 (WDR5) [17] (Figure 1B). Histone monoaminylation modifications represent a novel class of epigenetic regulatory mechanisms centered on dynamically reversible modifications mediated by TGM2 enzymes, rather than other enzymes in its family [29]. TGM2 plays a triple role in this process, acting as both the “writer” and “eraser” of modifications and catalyzing transamidation‐driven monoamine exchange [30]. When intracellular monoamine concentrations are sufficient, TGM2 acts as a “writer” to catalyze the formation of monoaminylation modifications. Conversely, when monoamines are deficient, TGM2's intrinsic hydrolase activity is activated, and it acts as an “eraser” to remove existing modifications [17, 31]. TGM2 exhibits unique enzyme kinetics. Its catalytic mechanism involves two key steps: first, an acyl transfer reaction, in which the cysteine residue (Cys277) in TGM2's active site nucleophilically attacks the γ‐carboxamido group of histone H3 Gln5, releasing an ammonia molecule and forming a thioester intermediate [32]. Second, a nucleophilic substitution reaction, in which monoamine neurotransmitters act as nucleophiles to attack this intermediate, creating a stable isopeptide bond and releasing the enzyme [33]. Moreover, TGM2‐mediated histone monoaminylation is primarily regulated by steric accessibility rather than by localized sequence features or pre‐existing PTMs [34]. The unique enzymatic property of TGM2 enables histone monoaminylation modifications to adjust in response to fluctuations in intracellular monoamine neurotransmitter concentrations, allowing the epigenetic state to respond dynamically to neuronal activity. Thus, given that TGM2 is involved in various cellular processes, including extracellular matrix organization, insulin release, and G‐protein signaling cascades, future studies should consider the impact of TGM2‐mediated dynamic modifications of epigenetic markers on gene expression [34].

Monoaminylation modifications catalyzed by TGM2 occur on both histones and non‐histone proteins via identical enzymatic mechanisms. First, substrate recognition necessitates conformational changes to expose cryptic glutamine residues (e.g., GTP‐bound RhoA revealing Q63 [35]), whereas histone modifications depend on disordered domain accessibility within chromatin‐permissive regions (e.g., H3 N‐terminal tails [15]). Second, microenvironmental control requires pathological stressors (e.g., oxidative stress or inflammation) to elevate cytosolic/extracellular Ca^2+^ concentrations (>100 µM) for TGM2 activation [36]. Furthermore, histone and non‐histone monoaminylation perhaps represent distinct biological response systems to differing physiological and environmental pressures. Histone monoaminylation is probably engaged in response to signals requiring broad genomic adjustments. By integrating into the chromatin template, it orchestrates widespread transcriptional reprogramming, thereby exerting a global and persistent influence on cellular physiological activity. Critically, histone serotonylation can mediate intergenerational effects, as evidenced by its role in placental signaling that links maternal environment to fetal neurodevelopment [37]. In contrast, non‐histone monoaminylation is more likely triggered by specific pathological or acute physiological stresses. It operates by modifying a limited set of target proteins, resulting in short‐term, localized adjustments to cell function [38, 39].

However, significant knowledge gaps remain regarding TGM2's substrate selectivity: why do specific brain regions tend to form particular types of monoaminylation modifications? How does TGM2 integrate local microenvironmental signals to determine its catalytic direction? These key questions remain unanswered. A deeper understanding of TGM2's catalytic mechanism and substrate selectivity is foundational to unraveling the complex functions of monoaminylation modifications.

## 3 The Role of Monoamine Transporters in Physiology and Disease

Accumulating evidence demonstrates that monoaminylation undergoes substrate‐driven interconversion at varying monoamine concentrations [17], indicating that dynamic shifts in local monoamine availability fundamentally regulate these modifications. Consequently, monoamine transporters, which precisely control the distribution of neurotransmitters across intracellular and extracellular compartments, represent pivotal modulators of monoaminylation [40, 41]. Despite comprehensive characterization of transporter physiology and pathophysiology, their direct mechanistic links to monoaminylation remain largely unexplored [42]. Therefore, elucidating how transporters govern monoamine bioavailability to regulate the incidence, specificity, and magnitude of modification remains a future research need. Established transporter targeting pharmaceuticals (e.g., inhibitors) provide potent tools for investigating and manipulating monoaminylation processes, enabling therapeutic repurposing strategies [43, 44, 45].

Monoamine transporters operate through two complementary systems. (1) plasma membrane transporters, primarily from SLC6/SLC22 families, are localized on neuronal or somatic membranes and mediate monoamine reuptake from synaptic clefts or extracellular spaces into the cytoplasm [40]. (2) Vesicular transporters (SLC18 family) reside on synaptic vesicle membranes and concentrate cytoplasmic monoamines into vesicles, achieving ∼10 000‐fold gradients, thereby facilitating their storage and activity‐dependent release [41, 46]. These systems cooperatively maintain spatiotemporal monoamine gradients, establishing a concentration‐sensitive equilibrium. Given the substrate dependence of monoaminylation, pharmacological or genetic disruption of this balance (i.e., altering regional monoamine concentrations) will directly impact modification efficiency and target specificity. Many transporters exhibit broad substrate promiscuity, with tissue‐ and disease‐specific dominance for particular monoamines [47]. Thus, defining context‐dependent transporter functions is essential for deciphering local monoamine dynamics and their functional consequences.

SLC6 plasma membrane transporters (SLC6A2, SLC6A3, and SLC6A4) orchestrate synaptic clearance of norepinephrine (NE), dopamine (DA), and serotonin (5‐HT) [48]. The serotonin transporter (SERT/SLC6A4) primarily regulates 5‐HT reuptake and its dysfunction correlates with diverse pathologies, including depression [49], cardiovascular regulation [50], metabolic homeostasis [51], and tumor immunomodulation [52]. SERT's pleiotropic effects via 5‐HT concentration control strongly implicate it in regulating 5‐HT‐dependent monoaminylation (e.g., serotonylation). Similarly, the dopamine transporter (DAT/SLC6A3) critically determines synaptic DA bioavailability [53]. Its dysregulation contributes to Parkinsonian neurotoxicity [54] and is subject to epigenetic control [55]. DAT regulates local DA concentrations in these cells, including lymphocytes and monocytes/macrophages [56], thereby modulating immune cell function [57]. As the principal gatekeeper of extracellular DA, modulation of DAT activity (via inhibitors or genetic approaches) likely regulates substrate availability for dopaminylation. Organic cation transporters (OCTs) mediate monoamine utilization in non‐neural tissues (e.g., liver, kidney, placenta) [58, 59]. Hepatic OCT1 (SLC22A1) influences drug responses and endogenous transport (including 5‐HT) [59, 60], and promotes colorectal cancer metastasis [61]. In astrocytes, OCT3 (SLC22A3) deletion reduces glial 5‐HT, impairing histone tryptophanylation, thereby causing GABAergic deficits and olfactory dysfunction [62]. This establishes a direct mechanistic link in which transporters regulate epigenetic modifications through local monoamine control, providing a paradigm for monoaminylation studies.

Vesicular monoamine transporters (VMAT1/SLC18A1, VMAT2/SLC18A2) utilize proton gradients to sequester cytoplasmic monoamines into vesicles [46, 63]. VMAT2 deficiency underlies neuropsychiatric disorders [63] and its imbalance with DAT (e.g., elevated DAT/reduced VMAT2 in ADHD) drives disease occurrence [64]. VMAT2 inhibitors (e.g., tetrabenazine [TBZ] [65], reserpine [66]) alter DA release by depleting vesicular stores or elevating cytoplasmic DA to induce reverse transport [67] by determining monoamine compartmentalization: vesicular storage (low modification potential) vs. cytoplasmic retention (high modification accessibility). Hence, VMAT2‐targeting compounds constitute ideal probes for interrogating the vesicular storage modification relationship. Moreover, clinically approved monoamine transporter inhibitors are widely used for neuropsychiatric disorders (e.g., depression, ADHD, tardive dyskinesia) [66, 68, 69], including selective agents (SERT inhibitors (sertraline [51], FLX, CIT [52]), DAT inhibitors (benztropine, β‐CFT, cocaine) [70]), multitarget agents (triple reuptake inhibitors (TRIs), DAT/NET dual inhibitors [68]) and natural derivatives (flavonoids, alkaloids with SLC6 inhibitory potential [68]). These pharmacological tools enable manipulation of compartment‐specific monoamine concentrations (extracellular, cytoplasmic, vesicular) across cellular, tissue, and organismal levels. Clinically validated agents (e.g., SSRIs [71], DAT [72]/VMAT2 antagonists [73, 74]) offer a practical means to perturb monoamine distribution. It is critical to recognize, however, that monoaminylation states are governed by a multifactorial interplay that extends beyond substrate availability. Key determinants include the writing activity and conformational regulation of TGM2, the potential for erasure or turnover mechanisms, the dynamics of local calcium microdomains, and the redox environment—all of which can directly influence TGM2 stability and catalytic function [75]. Consequently, while pharmacological perturbation of monoamine pools is a valuable approach to exploring the links between transporter function, substrate availability, and modification dynamics, robust causal attribution typically requires integrated experimental designs. These may combine transporter or vesicular manipulation with direct modulation of TGM2 activity, calcium signaling, or redox balance, coupled with orthogonal assays to verify changes in the modification state.

## 4 The Biological Implications of Histone Monoaminylation

**FIGURE 2:**
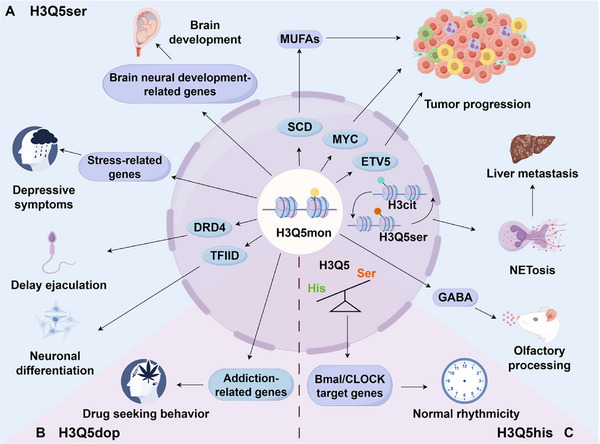
The roles of histone monoaminylation in disease pathogenesis. Histone monoaminylation regulates the development and progression of various diseases through distinct mechanisms. Panels illustrate the functional mechanisms of histone serotonylation (A), dopaminylation (B), and histaminylation (C), respectively, in different disease contexts. H3Cit: histone citrullination; GABA: Gama‐aminobutyric acid; MUFAs: monounsaturated fatty acids. We acknowledge the use of KingDraw software (KingDrawPc_V3.0.2.20) for drawing this figure.

Recent evidence has gradually uncovered the critical roles of histone monoaminylation—increasingly recognized as an emerging epigenetic mark highly enriched in cancer cells with significant gene regulatory functions—in a wide range of physiological processes in the nervous system and beyond, as well as their significant contributions to various diseases [76, 77, 78]. All three modifications are involved in neural function. Still, each focuses on specific processes, such as mood regulation, addiction, and circadian rhythms and their effects (promotion or inhibition) in disease, which are context dependent (Figure 2). A summary of the role of each modification in different physiological and pathological conditions is as follows (Table 1).

**TABLE 1:** Types and functions of histone and non‐histone monoaminylation.

Modification type	Source	Substrate proteins	Modification site	Biological function	Refs.
Serotonylation	Serotonergic neurons	H3	Q5	Low levels of H3K4me3Q5ser leads to depressive symptoms	[75]
Serotonylation	——	H3	Q5	Regulates olfactory behavior	[62, 79]
Serotonylation	——	H3	Q5	Accelerates the lip sensory recovery	[80]
Serotonylation	——	H3	Q5	Regulates offspring brain development	[37]
Serotonylation	Serotonergic neurons	H3	Q5	Promotes EPN tumorigenesis	[87]
Serotonylation	——	H3	Q5	Promotes pancreatic cancer progression	[88]
Serotonylation	Enterochromaffin cells	H3	Q5	Enhances CRC proliferation	[89]
Serotonylation	——	H3	Q5	Promotes HCC tumor progression	[90, 91]
Serotonylation	Neuroendocrine prostate cancer cells	H3	Q5	Leads to cancer metastasis	[92]
Serotonylation	——	H3	Q5	Delays ejaculation	[93]
Dopaminylation	Dopaminergic neurons	H3	Q5	Maintains transcription	[84]
Dopaminylation	Dopaminergic neurons	H3	Q5	Promotes heroin‐seeking behavior	[85]
Dopaminylation	——	H3	Q5	Affects synaptic plasticity	[86]
Histaminylation	Histaminergic neurons	H3	Q5	Regulates neural rhythmicity	[17]
Serotonylation	Pancreatic β cells, enterochromaffin cells	Rab3a/Rab27a	——	Promotes insulin granule release	[38, 39]
Serotonylation	CD8^+^T cells	GAPDH	Q262	Supportes antitumor immunity	[98]
Serotonylation	Enterochromaffin cells, colon cancer cells	mTOR	——	Maintains mTOR activation	[99]
Serotonylation	Enterochromaffin cells	RhoA	——	Promotes the carcinogenesis of colon cancer	[100]
Serotonylation	——	Rac1 or Cdc42	Q61 (Rac1)	Regulates dendritic spine plasticity	[82]
Serotonylation	Pulmonary endothelial cells	RhoA	Q63	Promotes proliferation of PA‐SMCs and platelet activation	[35]
Serotonylation	Intestinal cells	Ras Rac and Rho	——	Activates small GTPases	[101]
Serotonylation	——	SERCA2a	——	Promotes pulmonary vascular remodeling	[36, 102]
Serotonylation	Vascular smooth muscle cell	Smooth muscle α–actin	——	Modulates arterial contractility	[103]
Serotonylation	Mammary epithelial cells	RhoA	——	Mobilizes maternal bone calcium for milk synthesis	[104]
Serotonylation	Enterochromaffin cells	Neural surface proteins and fibronectin	——	Promotes thrombosis	[83]
Serotonylation, dopaminylation	——	Fibronectin	——	Regulates small GTPase activity	[30]
Dopaminylation	——	TPI1	Q65	Suppresses ferroptosis and promotes lung regeneration	[97]

### 4.1 Neuropsychiatric Disorders

Chronic environmental stress paradigms induce selective and progressive depletion of H3K4me3Q5ser within dorsal raphe nucleus serotonergic neurons, a change that correlates with stress vulnerability across multiple mammalian models and consistently observed in postmortem cortical tissues from patients with major depressive disorder [75], reflecting conserved epigenetic dysregulation spanning evolutionary lineages. This depletion disrupts the architectural integrity of permissive chromatin landscapes, which are necessary for maintaining transcriptional homeostasis in emotional processing circuits. Astrocyte‐specific modulation of H3Q5ser dynamics, mediated through activity‐dependent trafficking and subcellular localization of the neuromodulator transporter SLC22A3 across specialized glial compartments, constitutes a fundamental regulatory axis that directly coordinates GABA synthesizing enzyme expression profiles and vesicular neurotransmitter transporter networks within discrete neuroanatomical domains, thereby governing olfactory discrimination acuity thresholds and associative fear memory consolidation processes [62, 79]. In addition, exogenous serotonin promotes axonal growth in the lip via H3Q5ser following transection of the inferior alveolar nerve. This supplementation also slightly accelerated the recovery of lip sensation and was associated with elevated levels of H3Q5ser and axonal growth‐related molecules in the ipsilateral trigeminal ganglion [80]. Moreover, elevated serotonin levels in the placenta establish a unique biochemical milieu. Within this context, H3Q5ser is markedly enriched during critical developmental windows, a process that demonstrates stringent dependence on serotonin uptake by SERT. Genetic ablation of SERT profoundly diminishes genome‐wide H3Q5ser levels, consequently severely compromising the expression of cortical neurodevelopmental master regulators. Specifically, non‐neuronal H3Q5ser in the maternal placenta has been shown to regulate a placental transcriptome that, in turn, shapes the neurodevelopmental transcriptome in the fetal brain, thereby influencing the foundational architecture of neural circuitry [37]. This SERT‐dependent disruption of placental programming and the fetal brain transcriptome compromises early brain development [37, 81]. These developmental perturbations highlight the crucial role of monoaminergic‐epigenetic crosstalk in establishing the foundational architecture of neural circuitry. Beyond these chromatin‐centric mechanisms, non‑histone monoaminylation also directly regulates neuronal and cerebrovascular functions: serotonylation of Rac1 at glutamine‐61 (Q61) — downstream of 5‐HT2A/2C receptor activation — modulates synaptic plasticity through the activation of this small GTPase [82], while serotonylation of neural surface proteins and extracellular matrix components (e.g., fibronectin) contributes to thrombosis [83].

Within addiction neurocircuitry, protracted psychostimulant withdrawal triggers persistent striatal H3Q5dop accrual that sustains pathological transcription of addiction‐related immediate early gene networks through convergent molecular pathways involving ATP‐dependent chromatin architectural remodeling, high fidelity recruitment of bromodomain‐containing effector complexes to acetylated nucleosomal domains, stabilization of open chromatin conformations, and long‐term sensitization of mesolimbic dopaminergic reward pathways [84]. Opioid abstinence provokes accelerated ventral tegmental H3Q5dop accumulation, directly driving relapse vulnerability through dysregulated dopaminergic neurotransmission in cortico‐striatal glutamatergic projections via NMDA receptor hyperactivation [85], highlighting a convergent epigenetic vulnerability mechanism across distinct substance classes. Concurrently, early‐life adversity paradigms permanently reconfigure limbic epigenomes via stress‐induced H3Q5dop alterations, thereby establishing lifelong susceptibility to affective psychopathology through stable epigenetic reprogramming [86].

These findings position histone monoaminylation as a transformative, bidirectional interface that converts transient neurotransmitter signals into stable epigenetic memory modulating transcriptional programs that sculpt neural circuits and, thereby, shape neural circuit function and behavioral outputs. Future investigations must resolve spatiotemporal gradients of monoamine availability in specialized neural microdomains using advanced biosensor technology, develop cell‐type‐specific CRISPR‐based epigenetic editing platforms for causal manipulation, and establish definitive relationships between site‐specific modification dynamics and behavioral endophenotypes through longitudinal in vivo monitoring to enable next‐generation neuropsychiatric therapeutics targeting these precise molecular mechanisms. A comprehensive elucidation of crosstalk between monoaminylation and other epigenetic marks within defined neural populations will be crucial for mechanistically understanding the pathogenesis of psychiatric diseases.

### 4.2 Oncogenesis and Tumor Microenvironment

Monoaminylation exhibits profound context‐dependent oncogenicity through spatially and temporally compartmentalized mechanisms that operate across diverse epithelial, mesenchymal, and neural malignancies, exerting stage‐specific influences on tumor initiation, progression, and metastatic evolution. Serotonin exhibits paradoxical functionality across tissue boundaries: extrinsically suppressing ependymoma initiation through GABAergic neuron tumor synaptic inhibition within the specialized brain microenvironment, yet intrinsically promoting cell‐autonomous transformation via direct H3Q5ser‐mediated histone activation at oncogenic enhancer clusters and promoter regions, illustrating its microenvironment‐dependent duality [87].

Pancreatic ductal adenocarcinoma systematically exploits TGM2‐catalyzed H3Q5ser to transcriptionally upregulate lipid metabolism master regulators, including SCD1 and FASN, thereby driving malignant advancement through comprehensive metabolic network rewiring, altered membrane biophysical properties influencing receptor tyrosine kinase clustering, and growth factor signaling potentiation via specialized lipid raft microdomain organization [88]. This metabolic hijacking represents a targetable vulnerability in nutrient‐scarce microenvironments. Colorectal carcinomas strategically deploy H3Q5ser to induce inflammatory phenotypic conversion in stromal fibroblast populations, consequently amplifying tumor proliferative capacity through IL‐6/STAT3 axis hyperactivation, extracellular matrix proteolytic degradation via MMP overexpression, and immunosuppressive macrophage polarization through CSF1‐mediated recruitment within developing metastatic niches [89], effectively co‐opting stromal components to establish pro‐metastatic ecosystems. Similarly, hepatocellular carcinoma co‐opts this modification to potentiate super enhancer activity at chromatin domains marked by permissive histone modification signatures, consequently activating oncogenic transcription factor networks, including MYC and FOSL1, while accelerating disease progression through CDK‐mediated cell cycle checkpoint deregulation [90, 91]. Furthermore, cooperative interplay between H3Q5ser and histone citrullination promotes chromatin decondensation, ultimately driving neutrophil extracellular trap formation and liver metastasis in neuroendocrine prostate cancer [92].

This multifaceted oncogenic involvement underscores significant therapeutic opportunities for targeting modification‐specific molecular vulnerabilities. Prospective research should systematically map tumor microenvironmental monoamine flux gradients using spatially resolved metabolomics, characterize cancer cells, cancer‐associated fibroblasts, and immune cells’ epigenetic dependencies on specific monoaminylation marks through single‐cell epigenomic profiling, and validate innovative combination regimens integrating epigenetic modulators with neurotransmitter transporter inhibitors across malignancy spectra to overcome compensatory resistance mechanisms. Understanding the spatiotemporal evolution of these modifications within dynamically changing tumor ecosystems will be critical for developing stage‐adapted therapeutic interventions.

### 4.3 Circadian Regulation and Systemic Physiological Integration

H3Q5his constitutes an indispensable core component of mammalian circadian epigenetic oscillators that regulate sleep‐wake cycles and metabolic homeostasis and functions as a direct molecular transducer between core clock machinery and chromatin states. Its expression in tuberomammillary hypothalamic nuclei exhibits robust antiphasic oscillation relative to H3K4me3 dynamics, peaking during active wake phases under direct transcriptional governance of CLOCK‐BMAL1 heterodimeric complexes binding canonical E‐box enhancer elements [17]. Crucially, electrostatic repulsion from its protonated imidazole ring at physiological pH sterically hinders WD40 domain engagement within MLL methyltransferase complexes, thereby suppressing SETD1A/B‐mediated H3K4 methylation and downstream transcriptional activation of arousal‐promoting gene networks, including hypocretin and histidine decarboxylase [17], effectively functioning as a changeable regulator on wakefulness‐promoting transcription through charge‐mediated structural interference.

Beyond central circadian control, H3Q5his participates in systemic physiological integration through peripheral tissue oscillations. In reproductive neurophysiology, short‐acting serotonergic agents modulate ejaculatory control mechanisms by elevating spinal cord synaptic neurotransmitter concentrations, subsequently promoting H3Q5ser formation in lumbosacral autonomic nuclei to regulate ejaculation reflex arc excitability through transcriptional modulation of adrenergic receptor expression profiles [93], demonstrating the involvement of histone monoaminylation in peripheral autonomic reflex pathways beyond central nervous system functions. Given the distribution of these monoamines in diverse organs and tissues, their roles in other physiological and pathological contexts may be revealed in the future.

## 5 Multifaceted Functions of Non‐histone Monoaminylation in Normal Physiology and Pathophysiology

**FIGURE 3:**
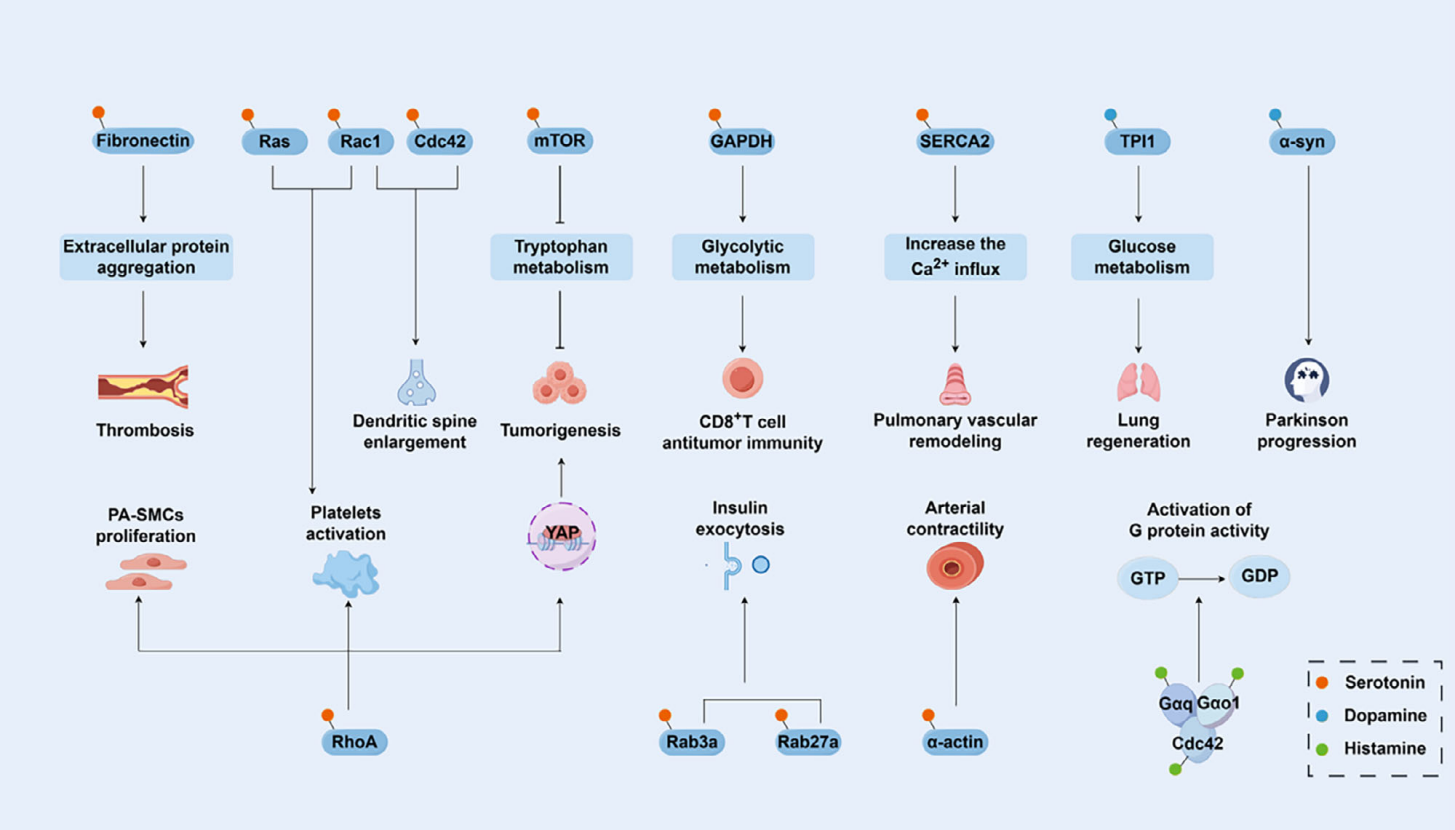
Non‐histone monoaminylation regulates diverse physiological processes. Monoaminylation of non‐histone proteins is involved in the regulation of multiple essential biological processes, including metabolic function, signal transduction, cardiovascular homeostasis, and immune regulation. We acknowledge the use of KingDraw software (KingDrawPc_V3.0.2.20) for drawing this figure.

As mentioned before, monoaminylation modifications are not only restricted to histones [94, 95, 96]. TGM2 catalyzes widespread monoaminylation of diverse non‐histone substrates, including metabolic enzymes, signal transduction proteins, and cytoskeletal proteins [22] (Figure 3). Understanding their functions is crucial for elucidating the role of monoamine neurotransmitters in shaping cellular physiology and pathology. Here, we systematically summarize the functional roles of non‐histone monoaminylation across different diseases and physiological systems (Table 1).

### 5.1 Metabolic Dysregulation and Cancer

Current studies have revealed that monoaminylation participates in disease formation and the maintenance of physiological homeostasis by regulating cellular metabolism. For instance, 5‐HT significantly elevates glucose‐stimulated insulin secretion by promoting the serotonylation of small GTPases Rab3a and Rab27a, which facilitates the release of insulin granules [38, 39]. In the progression of lung repair, TGM2‐mediated dopaminylation of triosephosphate isomerase 1 (TPI1) shifts its activity from phospholipid synthesis toward glycolysis. This metabolic reprogramming reduces lipid peroxidation, inhibits ferroptosis, and supports tissue regeneration, illustrating how a transient modification can rewire energy metabolism to meet dynamic cellular demands [97]. In the tumor microenvironment, serotonylation of GAPDH at Q262 in CD8^+^ T cells enhances glycolytic flux and augments their anti‐tumor effector function [98]. Conversely, serotonergic modification occurring in tumor cells can drive the progression of colorectal cancer. Inhibition of serotonin transporters (SERT) decreases the serotonylation level of mTOR, leading to its inactivation and tumor regression [99]. Furthermore, serotonylation of RhoA stabilizes YAP, thereby initiating a pro‐proliferative gene expression program that supports tumor growth [100]. Thus, monoaminylation orchestrates a complex, context‐dependent role in the tumor ecosystem. Together, these findings collectively establish non‐histone monoaminylation as a rapid, precise, and highly context‐dependent mechanism for metabolic rewiring. Its functional diversity originates from the selective modification of specific protein substrates, thereby profoundly influencing cell fate across scales from systemic physiology to the tumor microenvironment.

### 5.2 Cardiovascular Pathophysiology and Calcium Homeostasis

Monoaminylation is a critical regulator of cardiovascular function and calcium signaling, with its dysregulation underpinning several disease states. In pulmonary hypertension (PH), elevated serotonin drives serotonylation and constitutive activation of RhoA in pulmonary artery smooth muscle cells (PA‐SMCs), which in turn activates the Rho kinase pathway to stimulate PA‐SMC proliferation and vascular remodeling, a central pathogenic axis in this disease [35]. This pathway also contributes to platelet hyperactivation, linking vascular remodeling with thrombotic risk [101].

Beyond vascular remodeling, monoaminylation directly disrupts cardiac calcium handling. Sarcoplasmic/endoplasmic reticulum Ca^2+^ ATPase 2a (SERCA2a) is susceptible to serotonylation, particularly under hypoxia, resulting in reduced activity and consequent increases in intracellular calcium. This mechanism may regulate sinoatrial node pacemaking and pulmonary vein remodeling [36, 102]. Monoaminylation also directly modulates vascular tone. Serotonin directly alters vascular smooth muscle protein function via TGM2‐mediated modification, thereby modulating vascular contraction [103]. In lactation, mammary‐derived serotonin stimulates parathyroid hormone‐related protein (PTHrP) secretion to mobilize maternal bone calcium for milk synthesis, with concurrent elevation of transglutaminase activity [104]. Additionally, extracellular matrix proteins, such as fibronectin, undergo monoaminylation (with serotonin, dopamine, or norepinephrine) [30], which critically regulates small GTPase activity, platelet function, and other cell‐matrix‐dependent processes.

In summary, non‐histone monoaminylation constitutes a functionally diverse and widespread regulatory phenomenon. It orchestrates key physiological processes (insulin secretion, lactation, embryonic development, immunity) and contributes to disease development, including PH, cancer, cardiac arrhythmia, parkinson's disease (PD), and thrombosis.

## 6 Challenges and Advances in Defining the Biological Functions of Monoaminylation

**FIGURE 4:**
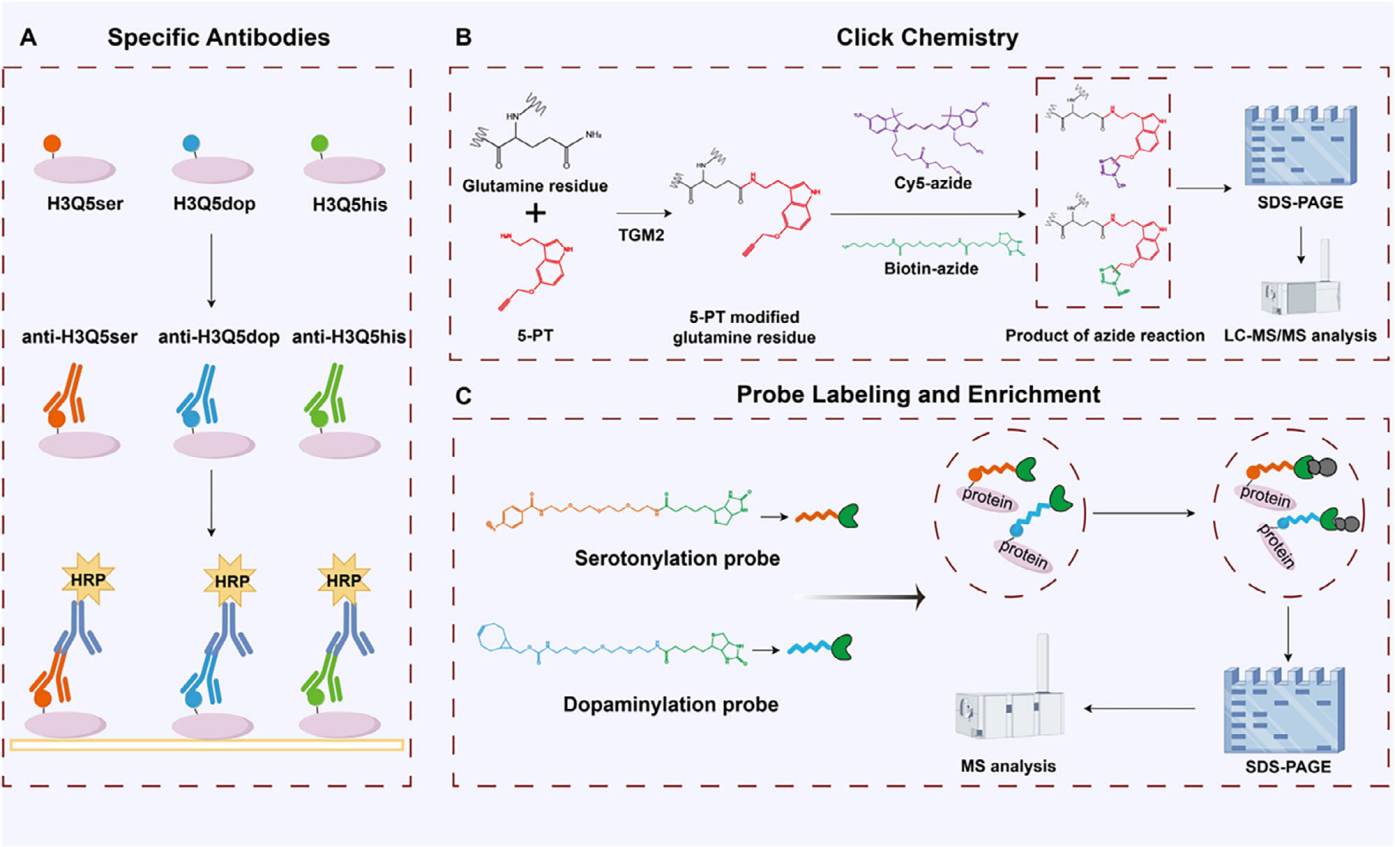
Current detection methods for protein monoaminylation. (A) Histone monoaminylation is commonly detected using modification‐specific antibodies. (B,C) Leveraging its structural similarity to serotonin, 5‐PT enables the detection of diverse serotonylated proteins via copper‐catalyzed azide‐alkyne cycloaddition (CuAAC) click chemistry (B). Biotin‐labeled probes have been developed for detecting protein serotonylation and dopaminylation (C) HRP: horseradish peroxidase. We acknowledge the use of KingDraw software (KingDrawPc_V3.0.2.20) for drawing chemical structures and Figdraw (www.figdraw.com) for assistance in creating schematic illustrations.

The lack of reliable and convenient detection assays hinders progress in monoaminylation research and limits the field. Existing methods include detection of monoaminylated protein levels using monoamine‐specific antibodies (Figure 4A), assessment of monoamine depletion via competitive analogs, and quantitative mass spectrometry [105]. However, existing assays suffer from inadequate differentiation between multiple monoamidations, poor specificity, and low sensitivity. Thus, to avoid cross‐reactivity with non‐monoaminylated proteins, a click chemistry detection method (Figure 4B) and chemical probes have been developed for different monoaminylation types to find modified proteins (Figure 4C).

5‐PT (5‐propargyltryptamine), an alkyne‐functionalized serotonin derivative, is incorporated into living cells and acts as a TGM2 substrate for serotonylation. This bioorthogonal tag enables the subsequent detection and enrichment of monoaminated proteins through CuAAC click chemistry using azide‐conjugated reporters [17, 98]. Besides, researchers developed a photoactive phenyl diazo‐biotin probe that specifically labelled and enriched protein serotonylation sites. This probe relies on a pH‐controlled chemoselective rapid azo coupling reaction (CRACR) [106].

For dopaminylation, parallel chemoproteomic strategies have been established. Two studies conducted systematic investigations about protein dopaminylation using structurally consistent chemical probes. The core of this methodology involves the oxidation‐controlled cyclooctyne‐1,2‐quinone cycloaddition reaction, which converts dopamine residues in proteins into dopamine quinones, followed by specific labeling and enrichment with BCN‐biotin probes. Notably, the dopamine quinone intermediate is itself highly reactive toward endogenous nucleophiles, particularly cysteine residues, forming stable Cys‐dopamine adducts. To specifically capture this non‐enzymatic yet functionally significant modification, complementary chemoproteomic strategies have been developed. For instance, probes targeting the cysteinyl‐dopamine moiety have been used to map its proteome‐wide landscape, revealing its potential protective role in neurodegenerative contexts, such as its modification of the Tau protein [107]. Because this adduct can also arise artifactually during sample preparation, rigorous controls are critical when applying these methods [108]. Collectively, both the quinone methide (QM)‐based probe and probes specific for cysteinyl‐dopamine adducts strategies are expanding the toolkit for mapping the diverse landscape of protein dopaminylation, each addressing distinct chemical forms of this modification.

Using this approach, both studies collectively constructed a global map of protein dopaminylation, encompassing both synaptic proteins [106] and histone and non‐histone proteins in tumor models [109]. These methods enable the specific detection of monoamine modifications in histones and non‐histone proteins, while effectively preventing cross‐reactivity between different modifications. Thus, developing novel specific probes holds promise for overcoming detection barriers, facilitating in‐depth investigations into the specific functions and mechanisms of monoaminylation modifications across various physiological and pathological contexts.

## 7 Conclusions and Perspectives

This article systematically reviews how monoamine neurotransmitters, such as serotonin, dopamine, and histamine, modulate the crosstalk among neural functions, physiological processes, and disease through protein monoaminylation mediated by TGM2. Given the pervasive neural innervation and the broad distribution of these neurotransmitters in systemic circulation and peripheral tissues, it is hypothesized that protein monoaminylation may play an essential role in both maintaining physiological homeostasis and facilitating disease progression.

However, research in this area still faces several critical challenges, which can be summarized as follows: (1) regarding modification types, whether additional neurotransmitters beyond those mentioned can undergo similar monoaminylation; (2) concerning catalytic enzymes, although TGM2 has been implicated in this process, it remains unclear whether other enzymes also contribute to monoaminylation; (3) in terms of substrate selection, how TGM2 perceives dynamic changes in intracellular monoamine concentrations and selects specific types of monoaminylation; (4) at the level of modification interplay, the regulatory network formed among different monoaminylation types remains poorly defined; (5) finally, regarding functional outcomes, the causal relationship between neural activity, monoaminylation events, and physiological or pathological phenotypes requires further clarification.

Addressing these challenges will require an integrated, multidisciplinary approach involving neurobiology, proteomics, chemical biology, and other relevant disciplines. Future breakthroughs will depend on multidisciplinary collaborative research. Chemical biology needs to develop more precise detection tools, structural biology should focus on conformational changes in the TGM2 nucleosome complex, neuroscience must dissect the behavioral effects of modifications at the circuit level, and clinical research needs to explore modification profiles in human samples. Only by integrating these multidimensional data can a comprehensive theoretical framework be built, spanning from molecules to behavior, ultimately enabling precise intervention strategies targeting the monoaminylation regulatory network to provide innovative therapies for oncology, developmental processes, and metabolic diseases.

## Author Contributions

Yiqi Zhao, Hongli Zhang, and Yating Yang drafted and conceptualized the manuscript, designed and organized figures and tables. Yan‐Dong Wang and Wei‐Dong Chen conceptualized, reviewed, and revised the manuscript and secured funding for this work. All authors have read and approved the review article.

## Funding

This work was supported by the Beijing Natural Science Foundation (Grant No. 7252088) to Y.‐D.W., the National Natural Science Foundation of China (Grant No. 82560575) to W.‐D.C., the National Key R&D Program of China (Grant No. 2021YFC2103900), and the National Natural Science Foundation of China (Grant No. 81970551) to Y.‐D.W.

## Declaration of Generative AI and AI‐assisted Technologies in the Writing Process

During the preparation of this work, the authors used Deepseek (version 3.2) solely to improve language. After using this service, the authors thoroughly reviewed and edited the content as needed and take full responsibility for the final publication.

## Conflicts of Interest

The authors declare no conflicts of interest.
